# The Linker Region Promotes Activity and Binding Efficiency of Modular LPMO towards Polymeric Substrate

**DOI:** 10.1128/spectrum.02697-21

**Published:** 2022-01-26

**Authors:** Aishwarya Srivastava, Pragya Nagar, Sumit Rathore, Nidhi Adlakha

**Affiliations:** a Synthetic Biology and Bioprocessing Laboratory, Regional Centre for Biotechnology, NCR-Biotech Cluster, Faridabad, Haryana, India; b Department of Biotechnology, All India Institute of Medical Sciences, New Delhi, India; Westerdijk Fungal Biodiversity Institute

**Keywords:** linker, carbohydrate-binding module, CAZymes, cellulose, *B. cinerea*

## Abstract

Lytic polysaccharide monooxygenases (LPMOs) mediate oxidative degradation of plant polysaccharides. The genes encoding LPMOs are most commonly arranged with one catalytic domain, while a few are found tethered to additional noncatalytic units, i.e., cellulase linker and carbohydrate-binding module (CBM). The presence of CBM is known to facilitate catalysis by directing the enzymes toward cellulosic polymer, while the role of linkers is poorly understood. Based on limited experimental evidence, linkers are believed to serve merely as flexible spacers between the structured domains. Thus, this study aims to unravel the role of the linker regions present in LPMO sequences. For this, we analyzed the genome of Botrytis cinerea and found 9 genes encoding cellulose lytic monooxygenases (AA9 family), of which *BcAA9C* was overexpressed in cellulose-inducible conditions. We designed variants of *fl*^LPMO^ (full-length enzyme) with truncation of either linker or CBM to examine the role of linker in activity, binding, and thermal stability of the associated monooxygenase. Biochemical assays predicted that the deletion of linker does not impact the potential of *fl*^LPMO^ for catalyzing the oxidation of Amplex Red, but that it does have a major influence on the capability of *fl*^LPMO^ to degrade recalcitrant polysaccharide substrate. Langmuir isotherm and SEM analysis demonstrated that linker domain aids in polysaccharide binding during *fl*^LPMO^-mediated deconstruction of plant cell wall. Interestingly, linker domain was also found to contribute toward the thermostability of *fl*^LPMO^. Overall, our study reveals that linker is not merely a spacer, but plays a key role in LPMO-mediated biomass fibrillation; these findings are broadly applicable to other polysaccharide-degrading enzymes.

**IMPORTANCE** The polysaccharide-disintegrating carbohydrate-active enzymes (CAZymes) are often found with multimodular architecture, where the catalytic domain is connected to an accessory CBM domain with the help of a flexible linker region. So far, the linker has been understood merely as a flexible spacer between the two domains. Therefore, the current study is designed to determine the role of linker in polysaccharide fibrillation. To conceive this study, we have selected LPMO as a model enzyme, as it is not only an industrially relevant enzyme but it also harbors a catalytic domain, linker region, and CBM domain. The present study highlighted the crucial and indispensable role of the linker region in mediating polysaccharide disintegration. Considering its role in binding, thermostability, and activity toward polysaccharide substrate, we propose linker as a potential candidate for future CAZyme engineering.

## INTRODUCTION

With wide-ranging global challenges and environmental concerns, there is a pressing need to transition from a fossil fuel-driven economy to a more sustainable bio-economy (1–4). In this regard, cellulose has emerged as an attractive and renewable source of alternative energy (5, 6). This crystalline cellulose can be depolymerized to fermentable residues with the help of an efficient cocktail of enzymes (7–9).

Recent advancement in the understanding of enzyme-based degradation has resulted in the development of efficient enzyme cocktails (10, 11). These hydrolytic consortia are essentially composed of fungal-origin, glycoside hydrolase enzymes, which are archived in a carbohydrate-active enzyme (CAZyme) database and divided into subfamilies based on their activities and active site residues (12, 13). Among these, endo-1,4-β-glucanases (cleave random internal bonds in cellulose chains), exo-1,4-β-glucanases (attack the reducing or nonreducing end of the cellulose polymer), and β-glucosidases (catalyze cellobiose-to-glucose conversion) (14–16) are observed to be major players in catalyzing biomass disintegration. Of late, it has been observed that complete cellulosic degradation requires the inclusion of lytic polysaccharide monooxygenases (LPMOs) in cellulase consortia (17, 18).

Discovered in 2010, lytic polysaccharide monooxygenases (LPMOs) are copper-dependent redox enzymes which require an external supply of electron donors for oxidative cleavage of polymeric substrate (19). This metalloenzyme inserts an oxygen atom at C1 or C4 of an intrachain cellobiosyl moiety, and this random internal cleavage in polymeric structure leads to the depolymerization of complex cellulose chain (20, 21). Based on their substrate specificity, LPMOs are archived in the CAZyme database in the AA (Auxiliary Activity) protein family under subfamilies AA9 to 11 and AA13 to 16: here, the AA9 family of proteins oxidatively cleaves cellulose (22), AA10 is involved in chitin disintegration, and AA13 mediates starch degradation (23).

Many LPMOs exist solely as catalytic modules, while a few are found tethered to additional noncatalytic modules, i.e., cellulase linker and carbohydrate-binding module (CBM). The presence of CBM facilitates catalysis by directing the enzymes toward cellulosic polymer; Chalak et al. (24) elucidated that the release of sugar was drastically reduced in CBM-truncated LPMO enzyme, indicating its role in assisting LPMO-mediated biomass fibrillation. The CBM is generally coupled to the catalytic domain of CAZymes via a linker region. These linkers are intrinsically disordered proteins, widely believed to be flexible spacers between the structured domains (25). However, it has only recently been realized that this linker region might support synergistic action of the catalytic and accessory domains. Linkers typically exist with variable length and little sequence conservation (26) and it is believed that each linker is tailored based on a specific structured domain type (27). There exists one common feature: bias toward specific amino acids such as Ser and Thr (27), which are generally glycosylated, contributing toward protection against proteolysis (28), expansion of the operating range of catalytic domain (29, 30), and/or binding to polymeric substrate (31). Additionally, cellulase linkers have been observed to improve temperature adaptation of cellulases, as seen in the case of Cel5G from P. haloplanktis (32). The cellulase linker has also shown to impart specificity to PoCel6A from Penicillium oxalicum (33). All these studies conclusively suggest that each cellulase linker has a unique, indispensable functional role in assisting the cellulose depolymerization process.

In spite of these findings, the impact of the linker region on the activity of modular LPMO has not been studied in detail. Courtade et al. (25) elucidated that the linker region acts as a connecting region between LPMO and CBM, which allows these domains to move independently of each other; however, other functional roles of the linker region in LPMO performance have not been revealed. Thus, considering the varied functionality of the linker region, the current study performed biochemical and biophysical analysis to understand its role in the activity and binding of LPMO toward polymeric substrate.

For this, we have mined the genome of phytopathogenic fungus Botrytis cinerea (*B. cinerea*) and identified a CBM containing AA9 LPMO (*BcAA9C*). The supplementation of full-length LPMO (*fl*^LPMO^) to a classical cellulase cocktail led to a 1.6-fold increase in glucose released from biomass disintegration, highlighting its importance in the saccharification of polysaccharide substrate. The enzyme possesses additional linker and CBM domains associated with catalytic domain. To understand the role of the linker and CBM domains associated with *fl*^LPMO^, we designed and expressed CBM- and linker-truncated enzymes in Pichia pastoris. Biochemical assays and biophysical techniques clearly indicated that the linker region facilitates binding and activity toward polymeric substrate. Furthermore, the presence of linker region was shown to contribute to the thermal stability of *fl*^LPMO^. Collectively, we have elucidated that the linker region present in *fl*^LPMO^ is crucial for its activity on polymeric substrate.

## RESULTS

### *B. cinerea* harbors multiple CAZymes associated with Auxiliary Activity.

**FIG 1:**
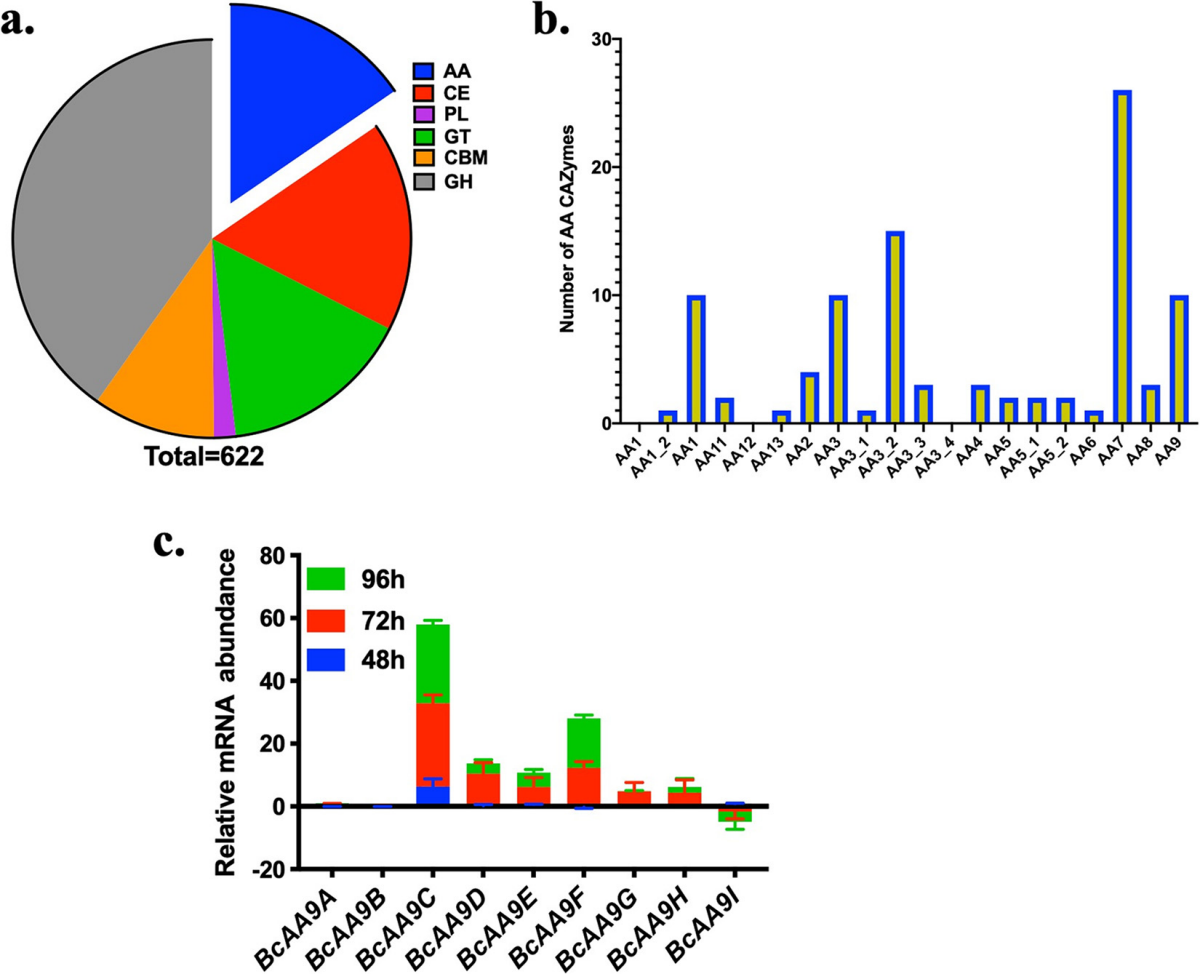
Polysaccharide monooxygenases encoded by Botrytis cinerea. (a) The genome sequence of *B. cinerea* 05.10 scrutinized for the presence of carbohydrate active enzymes (CAZymes) using dbCAN2 metaserver, which indicated the presence of 622 CAZymes. AA, Auxiliary Activity; CBM, carbohydrate-binding domain; PL, polysaccharide lyase; GT, glycosyl transferase; GH, glycosyl hydrolase; CE, carbohydrate esterase. (b) Bar graph indicates CAZymes belonging to different subfamilies of Auxiliary Activity, also revealing the presence of nine variants of polysaccharide monoxygenases which belong to the AA9 CAZyme family. (c) Relative mRNA abundance of nine *BcAA9* genes of Botrytis cinerea after 48 h, 72 h, and 96 h growth; *actin* was used as a housekeeping gene for RT-PCR. Error bar denotes standard deviation from three biological replicates.

The dbCAN analysis of publicly available *B. cinerea* genome indicated the presence of 622 carbohydrate active enzymes (CAZymes), and the those belonging to the Auxiliary Activity (AA) family accounted for almost 15% (Fig. 1a). CAZyme scrutiny, supported with the CAZy database (cazy.org), also suggested the presence of nine variants of cellulose lytic monooxygenases (AA9 family) (Table S2) in contrast to 7, 3, and 2 variants in A. niger, T. reesei, and T. cellulolyticus, respectively (Fig. 1b). Thus, we selected *B. cinerea* as the preferred fungal isolate for isolating enzymes particularly involved in oxidative degradation of cellulose.

To identify outperforming AA9 variants, we analyzed the expression of all nine homologues at the transcript level. qRT-PCR analysis suggested a 30-fold increase in the expression of *BcAA9C*, while *BcAA9D*, *BcAA9E*, and *BcAA9F* showed 4- to 8-fold increases and *BcAA9A*, *BcAA9B*, and *BcAA9I* did not show any expression compared with the *actin* control (Fig. 1c). Overall, *B. cinerea* secreted multiple cellulose lytic monooxygenases (AA9 family) into the medium (unpublished data), and among all the variants, *BcAA9C* was observed to express maximally. Therefore, we considered *BcAA9C*-encoded protein for further biochemical characterization and supplementation to augment biomass degradation by commercial enzyme cocktail.

### Design and *in silico* analysis of linker-truncated construct.

**FIG 2:**
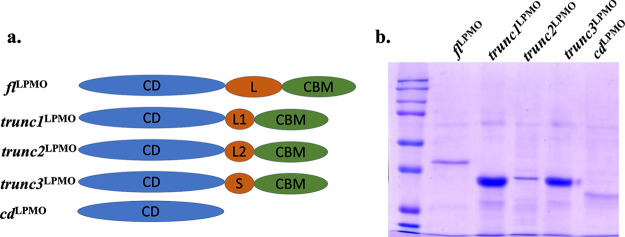
Design and purification of *fl*^LPMO^ and CD-CBM chimeric proteins. (a) Modular representation of *BcAA9C*-encoded LPMO variants, where *fl*^LPMO^ represents full-length protein (with catalytic domain, CD; linker, L; carbohydrate-binding module, CBM); *trunc1*^LPMO^, *trunc2*^LPMO^, and *trunc3*^LPMO^ represent CD-CBM chimeric proteins [where either linker is replaced with L1/L2-truncated linker sequence, or spacer sequence (S) is used to separate two domains]; and *cd*^LPMO^ represents catalytic domain without linker and CBM domains. (b) Purification of *fl*^LPMO^, *trunc1*^LPMO^, *trunc2*^LPMO^, *trunc3*^LPMO^, and *cd*^LPMO^ from Pichia pastoris KM71H, where SDS-PAGE gel indicates enzymes purified using Ni-NTA chromatography.

*BcAA9C* encodes a 32-kDa protein which harbors a CBM domain and linker sequence in addition to a catalytic domain (Fig. S1 in the supplemental material). Previous studies indicated the importance of the CBM domain in cellulase enzyme activity (34); however, the impact of the linker sequence has not been elucidated in detail. Therefore, to pin down biomass disintegration by monooxygenase protein and evaluate the impact of linker sequence and CBM domain on LPMO-mediated polysaccharide disintegration, we designed the following enzyme variants: full-length (*fl*^LPMO^), catalytic domain (*cd*^LPMO^), and CD-CBM (*trunc1*^LPMO^, *trunc2*^LPMO^, *trunc3*^LPMO^) (Fig. 2a).

To ensure that the replacement of linker with spacer sequence in the CD-CBM fusion constructs do not distort the stability of either domain, we screened the range of previously reported spacer sequences to ensure proper folding and stability in multidomain proteins (35). We analyzed the instability index of these chimeric proteins using the Expasy ProtParam tool. The instability index provides an estimate of the stability of proteins by analyzing the dipeptides, the occurrence of which is significantly different in stable versus unstable proteins (36). A protein with an instability index of greater than 40 is predicted as stable, while a value of less than 40 indicates that the protein may be unstable. Out of all the spacer sequences, CD-CBM fusion constructs with ELNYFQG, (EAAAK)_3_, and native linker truncations demonstrated instability indices of less than 40 (Table 1). Furthermore, the tertiary structures of both proteins were threaded using I-TASSER, and the secondary structures of fusion proteins were analyzed using GOR and Pdbsum, which showed that the secondary structures of the chimeric proteins were similar to that of the native protein, with a slight decline in the percentage of extended strands from 34.65% to 31.76%, in contrast to fusion protein with (EAAAK)_3_, which demonstrated a reduction in alpha helix with reduced disulfide bonds (Fig. S2). Therefore, we selected ELNYFQG as a suitable spacer sequence and other native linker truncations to construct chimeric CD-CBM fusion constructs.

**TABLE 1:** The physiochemical properties of *fl*^LPMO^ and chimeric constructs

No.	Spacer sequence	Instability index
1	Native linker	38.38
2	ELNYFQG	39.97
3	(GGGGS)_2_	42.81
4	(GGGGS)_3_	43.59
5	(Gly)_6_	42.51
6	(Gly)_8_	43.22
7	—^*a*^	41.99
8	(EAAAK)_3_	38.97
9	TIPGPTPFVCGAAQSTAK^*b*^	39.87
10	TIPGPTPFVCGAAQSTAKSSSTSTAKPTSTSTLSTSTVTKTSSS^*c*^	39.98

a Indicates no linker.

b Linker truncation 1 (L1).

c Linker truncation 2 (L2).

The tertiary structures of chimeric constructs and *fl*^LPMO^ were threaded based on PDB templates such as 4eirA, 6rs6A, 5foh, 6rs6, 4eir, 5x39A, and 1cbhA, and models with highest confidence scores were selected to estimate structural similarity between two constructs. Structure superimposition using Swiss-PdbViewer indicated high similarity, with a RMSD (root mean square deviation) of −0.297. I-TASSER also predicted that the ligand binding site was conserved (H85, H1, and Y170) and unperturbed in the chimeric construct. Furthermore, the models were subjected to energy minimization by SPdbV (Swiss-PdbViewer), which displayed that the chimeric constructs (*trunc1*^LPMO^, −8727.556 KJ/mol; *trunc2*^LPMO^, −8178.429 KJ/mol; *trunc3*^LPMO^, −9987.863 KJ/mol) and the full-length construct (−7277.997 KJ/mol) had high structural stability.

### Purification and biochemical characterization of *fl*^LPMO^, *trunc1*^LPMO^, *trunc2*^LPMO^, *trunc3*^LPMO^, and *cd*^LPMO^.

**FIG 3:**
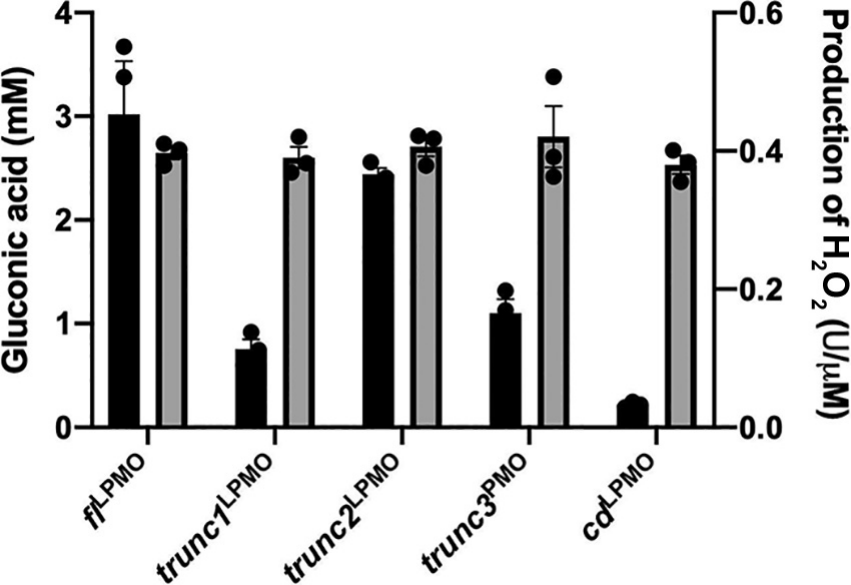
Biochemical characterization of *fl*^LPMO^, *cd*^LPMO^, *trunc1*^LPMO^, *trunc2*^LPMO^, and *trunc3*^LPMO^. Specific activity of full-length and truncated monooxygenases on complex and simple substrate. Gray bar indicates Amplex Red assay using 1 µM protein, where *y* axis indicates µmol of H_2_O_2_ released per unit of time per µM protein; black bar indicates LPMO-assisted degradation of PASC, where 8 g/L PASC was treated for 48 h at 45°C with 10 µM copper-saturated LPMO enzymes and 2 mM ascorbate. The hydrolysis of oxidized products was performed at 40°C for 24 h using 5 units of β-glucosidase, and the release of gluconic acid was estimated using HPLC equipped with a photodiode array (PDA) detector. Error bars represent standard deviation of three independent experiments.

All proteins were expressed in a Pichia pastoris system and purified using Ni-NTA affinity chromatography (Fig. 2b). The purified enzymes were assayed against Amplex Red, and the release of H_2_O_2_ per unit of time was monitored. Interestingly, activity in the truncated constructs was broadly unaffected, which again confirms the integrity of the catalytic domain across all variants (Fig. 3).

However, when assayed on a polysaccharide substrate, PASC (phosphoric acid swollen cellulose), a decline in activity was observed in all *trunc1*^LPMO^, *trunc2*^LPMO^, *trunc3*^LPMO^, and *cd*^LPMO^. Precisely, 8 g/L PASC was incubated with 10 µM copper-saturated LPMO enzymes and the oxidized products were hydrolyzed using glucosidase to release gluconic acid, which was monitored using high-performance liquid chromatography (HPLC) (Fig. S3). The experimentation results indicated that truncation of the CBM domain resulted in a 93% decline in cellulose oxidation activity, whereas linker-truncated constructs demonstrated a 34 to 79% reduction in activity, compared to that of the full-length construct (Fig. 3). Precisely, *trunc2*^LPMO^ with a longer stretch of native linker sequence displayed higher activity than the other two constructs, confirming the importance of complete linker sequence in mediating polysaccharide degradation. Overall, the results clearly indicate the essential roles of linker and CBM in enhancing the rate of oxidative degradation of polysaccharide substrate.

### Influence of accessory domains on binding to polysaccharide substrate.

**FIG 4:**
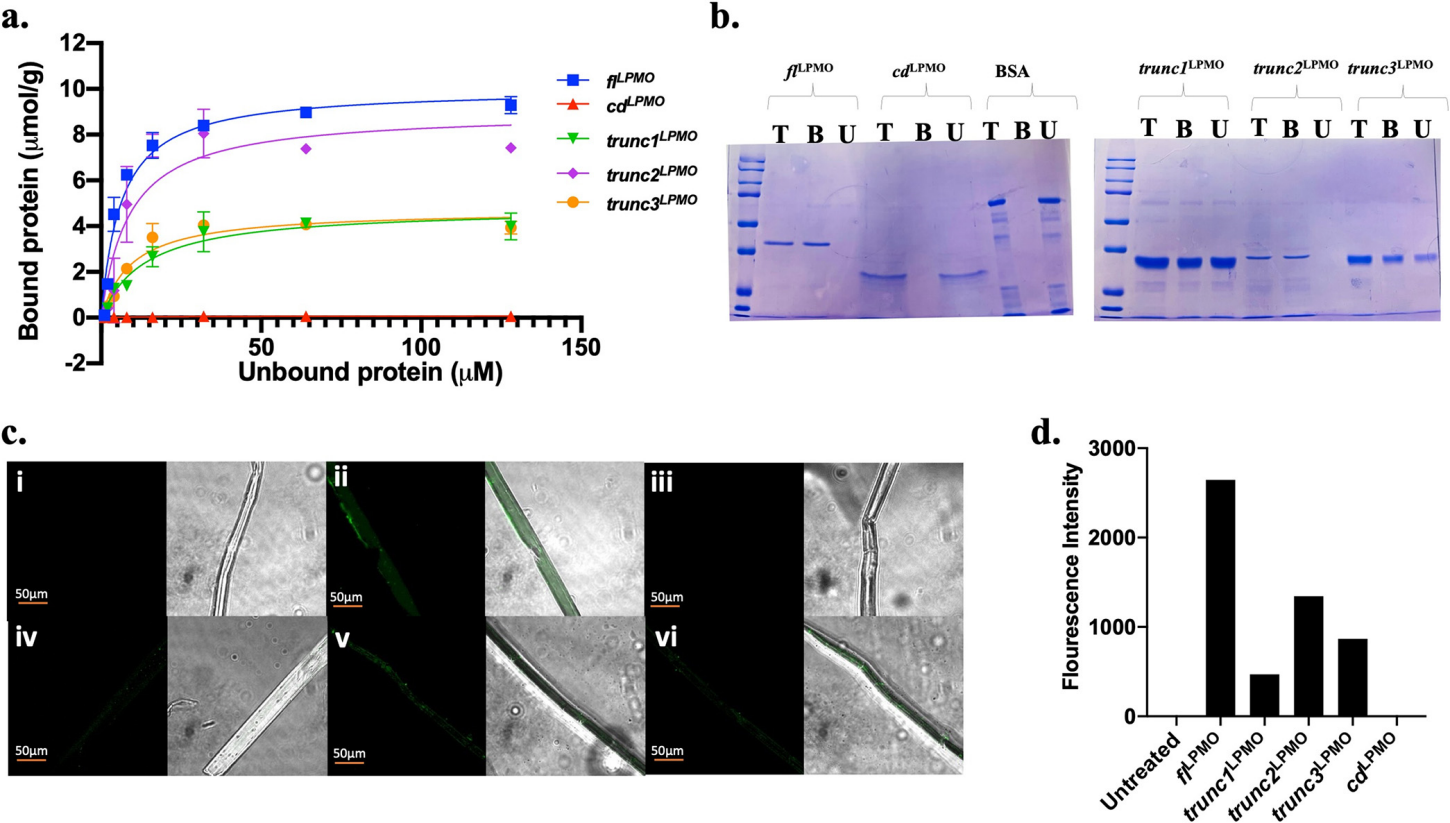
Influence of linker on binding polysaccharide substrate. (a) Binding isotherms for full-length (*fl*^LPMO^), CD-CBM (*trunc1*^LPMO^, *trunc2*^LPMO^, *trunc3*^LPMO^), and CD (*cd*^LPMO^), where specific binding of protein was calculated by the following equation: [total protein-free protein (post binding)]/g biomass. (b) Representation of binding experiment where 120 µM protein was allowed to bind 2 mg biomass for 12 h at 4°C in 250 µL reaction and total protein (T), Bound fraction (B), and Unbound fraction (U) were loaded onto SDS-PAGE gel (14%). BSA was used as a negative control (c) The protein-bound biomass was analyzed for protein binding using confocal microscopy. The protein-bound biomass fraction was probed with anti-His primary antibody and anti-IgG-FITC secondary antibody to visualize specific binding using confocal microscopy, where the binding of different constructs is represented by (i) BSA, (ii) *fl*^LPMO^, (iii) *cd*^LPMO^, (iv) *trunc1*^LPMO^, (v) *trunc2*^LPMO^, and (vi) *trunc3*^LPMO^. (d) FITC fluorescence intensity averaged for nine images indicates quantitative binding of LPMO constructs.

Here, we aimed at exploring the role of linker sequence in increasing proximity of *fl*^LPMO^ to recalcitrant cellulose. For this, the binding strengths of *fl*^LPMO^, *trunc1*^LPMO^, *trunc2*^LPMO^, *trunc3*^LPMO^, and *cd*^LPMO^ were tested against polysaccharide substrates. Each construct was evaluated at concentrations ranging from 0 to 150 µM for binding to pretreated biomass (Fig. 4a). The curve between bound protein (µmol/g) and amount of free protein (µM) was used to compute partition coefficients, Kr (B_max_/*K_d_*). The experimental results indicated that the deletion of linker led to a 1.5- to 7-fold reduction in binding, whereas removal of CBM completely aborted binding to insoluble substrates. On Avicel, the Kr value followed a similar trend as on pretreated biomass (PTB); Kr(*fl*^LPMO^) > Kr(*trunc2*^LPMO^) > Kr(*trunc3*^LPMO^) ∼ Kr(*trunc1*^LPMO^) > Kr(*cd*^LPMO^) (Table 2).

**TABLE 2:** The partition coefficients (Kr = Bmax/*K_d_*) for LPMO variants from the Langmuir isotherm

LPMO variant	*K_d_*	B_max_	B_max_/*K_d_*
*fl* ^LPMO^	6.218	10.02	1.61
*cd* ^LPMO^	18.74	0.07915	4.22 × 10^−3^
*trunc1* ^LPMO^	22.87	5.283	2.31 × 10^−1^
*trunc2* ^LPMO^	8.844	8.997	1.07
*trunc3* ^LPMO^	20.66	5.081	2.45 × 10^−1^

As detailed in Methods section; the fractions of bound, unbound, and total protein for each construct were run on SDS-PAGE gel; in concordance with depletion isotherm, the binding gel showed that *fl*^LPMO^ exhibited complete binding to PTB, and linker-truncated constructs demonstrated reduced binding to polysaccharide substrate, showing that linker plays a crucial role in binding to polysaccharide substrate (Fig. 4b).

Further, confocal microscopy of biomass confirmed that the binding of *fl*^LPMO^ declines with truncation of the CBM and linker domains (Fig. 4c). We also observed that deletion of the carbohydrate-binding module led to complete loss of the capability of *fl*^LPMO^ to bind recalcitrant biomass (Fig. 4d). Overall, the presence of the linker and CBM domains was found to enhance the binding of LPMO to insoluble and crystalline substrates.

### Influence of linker on thermostability of *fl*^LPMO^.

**FIG 5:**
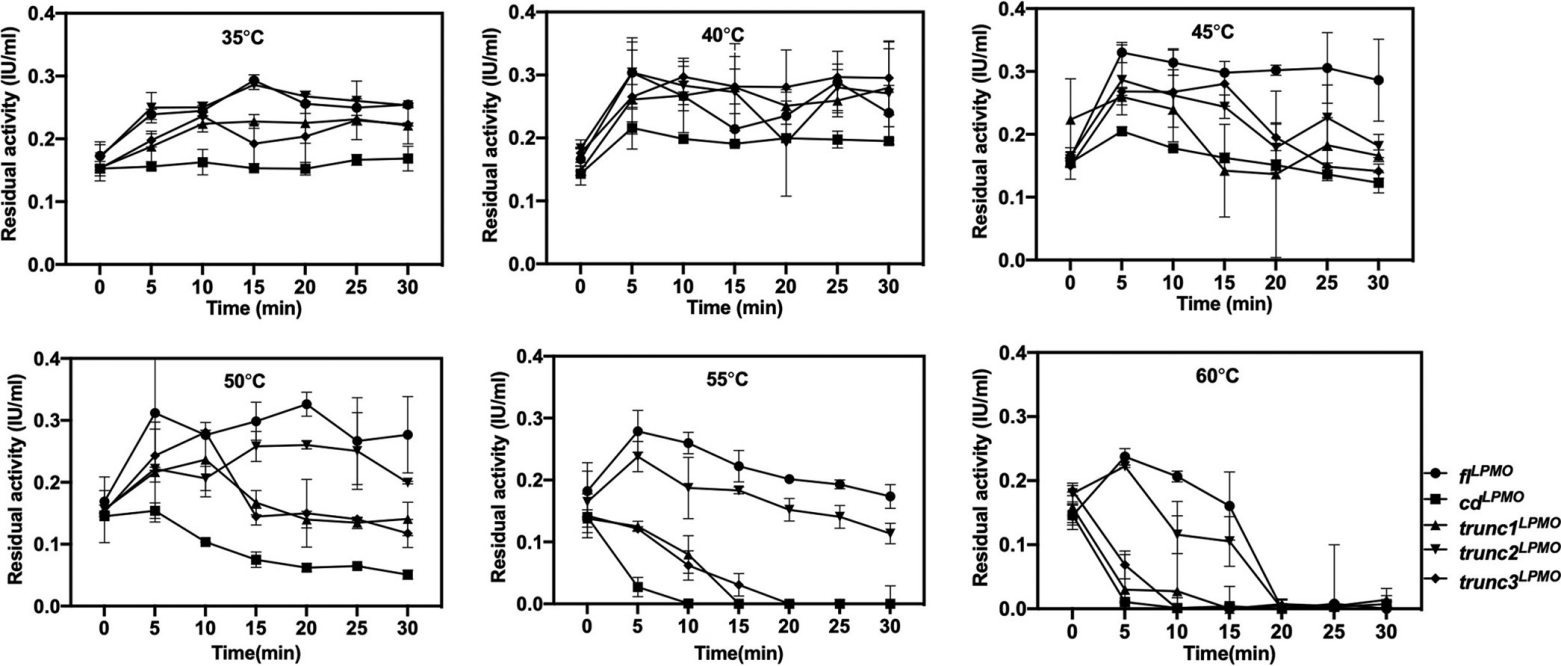
Effect of linker on thermostability of recombinant polysaccharide monooxygenase. LPMO activity of full-length (*fl*^LPMO^), CD-CBM (*trunc1*^LPMO^, *trunc2*^LPMO^, *trunc3*^LPMO^), and CD (*cd*^LPMO^) construct was tested at a temperature range of 35 to 60°C for 30 min. The enzymes were preincubated at 35°C, 40°C, 45°C, 50°C, 55°C, and 60°C for 5 to 30 min and immediately tested for residual monooxygenase activity using a reference Amplex Red assay, which estimates µmol of H_2_O_2_ released per unit of time. Error bars represent standard deviation of two independent experiments.

The effect of the linker region on the thermostability of *fl*^LPMO^ was investigated by measuring the residual activities of *fl*^LPMO^, *cd*^LPMO^, *trunc1*^LPMO^, *trunc2*^LPMO^, and *trunc3*^LPMO^ at various temperatures. The monooxygenase activities of all constructs remained constant for the initial 30 min at a temperature range of 35 to 45°C. At 50°C, an approximately 65% decline in activity was observed within 30 min in the *trunc1*^LPMO^, *trunc3*^LPMO^, and *cd*^LPMO^ constructs, whereas activity was still maintained in *fl*^LPMO^ and *trunc2*^LPMO^. With a 5-degree increase in temperature, complete loss of activity was observed in less than 15 min in the *trunc1*^LPMO^, *trunc3*^LPMO^, and *cd*^LPMO^ constructs, whereas the *fl*^LPMO^ and *trunc2*^LPMO^ constructs demonstrated 46% and 65% declines in activity in 25 min, respectively. Furthermore, the presence of linker increased the thermostability of monooxygenase enzyme by 10 min at 60°C (Fig. 5). By and large, linker imparts thermostability to its associated monooxygenase catalytic domain.

### Effect of LPMO on degradation of PTB.

Recent studies have reported the role of LPMO in accelerating the degradation of recalcitrant biomass (37). However, the importance of the linker and CBM domains in assisting LPMO-mediated disintegration of cellulosic biomass is poorly understood. Here, we have analyzed the effect of LPMO on disintegration of recalcitrant biomass using biophysical and biochemical parameters.

Scanning electron microscopy (SEM) was used to observe surface changes in pretreated biomass post-exposure to 10 µM *fl*^LPMO^, *cd*^LPMO^, or CD-CBM fusion constructs. Figure S6i and iii show the negative and positive controls, respectively; Fig. S6i represents the surface morphology of untreated pretreated biomass, whereas Fig. S6iii represents the morphology of commercial enzyme-treated biomass. SEM analysis of the *cd*^LPMO^ (Fig. S6ii)-, *trunc1*^LPMO^-, and *trunc3*^LPMO^ (Fig. S6v and S6vii)-treated samples displayed marginal changes in surface architecture, while the *fl*^LPMO^ (Fig. S6iv) and *trunc2*^LPMO^ (Fig. S6vi) microscopy images display increased surface roughness compared to the control, indicating that both the linker and CBM domains are crucial for the binding and hydrolysis of plant biomass structure.

Furthermore, we performed a biochemical assay to measure the increase in glucose release upon LPMO-assisted biomass degradation. As anticipated, the degradation followed the same trend as for SEM: *fl*^LPMO^ > *trunc2*^LPMO^ > *trunc3*^LPMO^> *trunc1*^LPMO^> *cd*^LPMO^ (Fig. S4).

### Synergistic depolymerization of PASC by LPMOs and classical cellulase cocktail.

**FIG 6:**
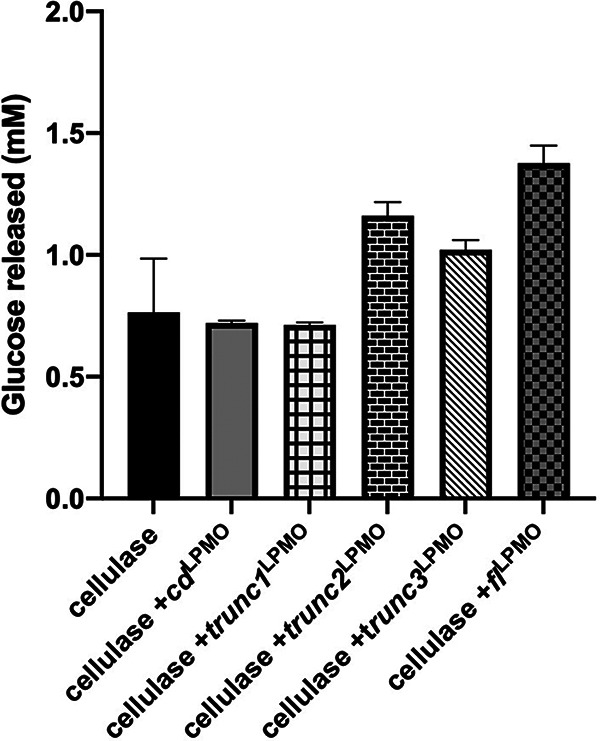
Synergistic action of LPMOs and cellulases. Eight g/L PASC was incubated with 0.5 FPU cellulase (C2730, Sigma), 10 µM copper-saturated *fl*^LPMO^, *trunc1*^LPMO^, *trunc2*^LPMO^, *trunc3*^LPMO^, or *cd*^LPMO^, and 2 mM ascorbate for 72h at 45°C, and the release of glucose was measured using HPLC equipped with a Hi-Plex Ca column. Error bars indicate standard deviations from three independent experiments.

LPMOs are known to augment the degradation of polysaccharide substrates by classical cellulase cocktail. Therefore, we evaluated the contribution of *fl*^LPMO^ toward depolymerization of crystalline polysaccharide substrate, PASC. Furthermore, we also studied importance of the linker and CBM sequences in *fl*^LPMO^-assisted cellulose disintegration. For this, the glucose yield generated by the combined action of cellulase and the *fl*^LPMO^, *cd*^LPMO^, or CD-CBM fusion constructs was analyzed using HPLC equipped with a HiPlex-Ca column (Fig. S5). The addition of *fl*^LPMO^ led to a 1.6-fold increase in glucose released over time, whereas the addition of *trunc2*^LPMO^ and *trunc3*^LPMO^ led to mere 1.4- and 1.1-fold increases, respectively; no change was observed in the CBM-truncated or linker-truncated constructs with shorter stretches of linker sequence (Fig. 6). The results also highlighted the importance of the complete linker region in LPMO-assisted biomass disintegration.

## DISCUSSION

Cellulose lytic monooxygenases belong to the AA9 family of CAZymes, which catalyze oxidative degradation of cellulose. LPMOs are reported to augment biomass disintegration by classical hydrolytic enzyme cocktail (38), and therefore, persistent efforts are being led to discover newer LPMOs (39–41). These LPMOs mainly interact with polysaccharide substrate via accessory domains, i.e., carbohydrate-binding module (CBM) (24, 25). Generally, these binding modules are tethered to the catalytic domain via linker regions, which are considered to be flexible hinges between two structured domains (25); however, the other functional role of the linker region in LPMO performance has not yet been revealed. Here, we aim to unravel the role of the linker region in the activity, binding, and thermostability of its associated protein.

For this, we have mined the genome of the fungal phytopathogen *B. cinerea* for the presence of cellulose lytic monooxygenases and identified *BcAA9C* as one of the majorly expressed monooxygenases. The *BcAA9C*-encoded, 32-kDa *fl*^LPMO^ is a protein with a 5.2-kDa linker region and a 3.2-kDa CBM domain. To understand the importance of linker, we designed linker- and CBM-truncated constructs. Briefly, to prune the linker domain, we replaced the linker sequence with a 21-nucleotide helical spacer sequence to allow independent folding of the catalytic and carbohydrate-binding domains. Furthermore, we designed two additional fusion constructs with truncation of the native linker sequence. We analyzed the physicochemical properties of a CD-CBM chimeric construct to ensure that the switch of linker domain to spacer sequence did not disturb the folding of the protein. Biochemical assays also suggested that the fusion and the native protein displayed similar potential to oxidize Amplex Red (Fig. 3) and that the active center is unperturbed, suggesting that the constructs are ideal to study the role of the linker present in the LPMO sequence.

The oxidative degradation of PASC declined by 93% with CBM deletion, whereas linker truncations resulted in a 34 to 79% reduction, suggesting the importance of the linker and CBM sequences in biomass degradation. Langmuir depletion isotherm also showed that removal of the linker domain resulted in 7-fold reduced binding efficiency to polysaccharide substrate, emphasizing the role of the linker domain in binding to cellulosic chain (Fig. 4a). Using confocal microscopy, we confirmed the role of linker in binding, as a linker-free, chimeric *cd-cbm*^LPMO^ protein demonstrated significantly less binding than *fl*^LPMO^. Furthermore, it is evident from confocal and binding experiments that a longer stretch of native linker in CD-CBM fusion constructs ensures improved binding, confirming the crucial role of linker in assisting binding to cellulose. This was in concordance with Badino et al. (42) who suggested that moderate modification of linker sequence in cellobiohydrolase could perturb its substrate affinity. Another study by Srisodsuk et al. (43) also demonstrated that, at high enzyme concentration, interdomain linker plays a crucial role in supporting polysaccharide-substrate binding. One possible reason for its role in mediating interaction between substrate and catalytic domain could be its typical glycosylation pattern. Sammond et al. (27) preferentially studied linkers connecting glycosyl hydrolase family 6 or 7 to carbohydrate binding modules 1 or 2, and identified that the linker sequence displays very little sequence conservation and significant amino acid bias toward serine/threonine, which serves as potential hot spots for O-glycosylation. These O-glycosylation sites were shown to expand the operating range of enzymes (44). In addition, glycosylation plays myriad biological roles in providing proteolytic stability to cellulose-degrading enzymes (28). Following these studies, we performed prediction of glycosylation patterns in *fl*^LPMO^ using the Net OGly 4.0 server (45), which distinctly revealed that in a complete protein sequence, only the linker region is heavily O-glycosylated.

Along with binding, we demonstrated the role of linker region in imparting thermal stability to the catalytic domain. In accordance, Miao et al. (46) has shown that appending the linker region and CBM1 to GH10 xylanase increases its thermostability. The group has also shown that deletion of the linker region led to a marked decline in enzyme stability. Contrary to this, Sonan et al. (32) demonstrated a role of linker in cold adaptation, indicating that each cellulase linker has a unique, indispensable functional role in imparting stability; therefore, it was important to analyze the linker associated with *fl*^LPMO^. Our study highlighted the importance of linker in increasing the thermostability of associated cellulose monooxygenase (Fig. 5).

The goal of scrutinizing any new CAZyme is to improve existing cellulase cocktail for efficient biomass deconstruction. Therefore, we analyzed the roles of *fl*^LPMO^, *trunc1*^LPMO^, *trunc2*^LPMO^, *trunc3*^LPMO^, and *cd*^LPMO^ in biomass fibrillation using SEM, which indicated that the linker and CBM domains boost degradation of pretreated biomass. Overall, our experimental results suggest that linker region is as crucial as catalytic domain in dismantling polysaccharide biomass and, therefore, that engineering the linker region may also constitute an efficient method for improving cellulase activity.

## MATERIALS AND METHODS

### Strains and media.

The fungus Botrytis cinerea was taken from the Indian Type Culture Collection, India (accession no. −6192). *Botrytis* sp. (1 × 10^6^ spores/mL) was inoculated in complex medium (Soya Peptone 24 g, ammonium sulfate 3.12 g, CaCl_2_.2H_2_O 0.05 g, KH_2_PO_4_ 5.9 g, yeast extract 0.05 g per liter of medium) containing 21.4 g/L wheat bran and 24 g/L Avicel and incubated on a shaker for 5 days at 22°C, and samples were withdrawn at different time points for expression studies.

Escherichia coli strain DH5-Alpha was used for cloning genes in pPICZ(alpha)A vector, while protein expression was performed in Pichia pastoris KM71H using the standard instructions in an EasySelect Pichia Expression kit (Invitrogen).

### Pretreatment of wheat straw.

Sodium hydroxide-treated wheat straw was prepared using the protocol developed by Xu et al. (47). Briefly, wheat straw was treated with 1% NaOH for 12 h at 50°C under continuous agitation. The treated biomass was then washed extensively with distilled water until it reached a neutral pH. Excess water in pretreated biomass (PTB) was removed by keeping it in an oven at 70°C for 5 h. The cellulosic and hemicellulosic content in PTB was analyzed using a standard protocol (48) (Table S1 in the supplemental material).

### Functional annotation.

Analysis of carbohydrate active enzymes (CAZymes) was performed using proteins downloaded from the Botrytis cinerea genome sequence (NCBI GenBank Assembly accession no. GCA_000143535.4). The dbCAN2 metaserver was used to predict the list of CAZymes (49). The Hidden Markov model algorithm (50) was used in HMMER 3.0 (51) to retrieve CAZYmes with an E value of 10^−5^.

### RNA extraction and qRT-PCR.

*B. cinerea* (1 × 10^6^ spores/mL) was inoculated in Avicel- and wheat bran-containing complex medium and mycelium was recovered after 48 h, 72 h, and 96 h cultivation. The mycelium was crushed in liquid nitrogen using a mortar and pestle, and 100 mg mycelium powder was utilized for RNA extraction using the manufacturer’s instructions (Qiagen Plant RNA extraction kit). RNA samples were quantified using NanoDrop (Thermo Fisher) and 2 µg RNA was used for cDNA synthesis using the manufacturer’s instructions (High Capacity cDNA Reverse Transcription Kit, Thermo Fisher Scientific).

For real-time PCR, primers for nine BcAA9 genes were designed using IDT software considering amplification of 160 bp amplicon and *T_m_* = 60°C (Table 3), and *actin* was used as a housekeeping gene. Two μL of cDNA was used as the template and the reaction mix was prepared with 10 µL SYBR Green Master Mix (Takara Bio), 0.8 µL Primer1, 0.8 µL Primer2, 0.4 µL ROX reference dye, and 7.44 µL nuclease-free water. After reaction assembly, an Applied Biosystems real-time PCR machine was used to perform qPCR with the following conditions: initial denaturation at 95°C for 2 min; 40 cycles of 95°C for 15 s, 55°C for 15 s, and 72°C for 15 s; and final extension of 72°C for 2 min. Raw data were analyzed using the ΔΔCt method and results were plotted in GraphPad Prism 8.0. All experiments were performed in two technical and three biological replicates, and standard deviation in the fold change was calculated accordingly.

**TABLE 3:** Primer sequences for RT-PCR

Gene	Forward primer	Reverse primer
*BcAA9A*	GCTAGCAATGATCTACGAT	GACCCTGATGATAAACCTG
*BcAA9B*	GTATTGGTTCATGGTTCAAG	TGAACGAAGCAAGTATTCAC
*BcAA9C*	GAATACGTCGCCTGCAATG	ATCATTTGCAGGTGTGCTG
*BcAA9D*	CGTTCTTTCACCATTCCATC	CAGACCCAGTTACGCGTAG
*BcAA9E*	CAGATCCCTACTATGCTACA	GCTGTGAACCAACAATACC
*BcAA9F*	CACAACCCGATTCTATCGG	GCGTTGGTAGCCTCCTTAG
*BcAA9G*	CAATCGAACGGTTTCATTGC	CCATTGCAAGTTAACCTCAC
*BcAA9H*	GTGTGTTATGATTGTGCGC	CCGTCTTAACAATCACAGG
*BcAA9I*	GCTGGCTGATACCTAAAGAT	CTTTAACAGGAGCCTCGATC
*Actin*	GAATACGTCGCCTGCAATG	ATCATTTGCAGGTGTGCTG

### Expression of full-length, truncated, and chimeric LPMO constructs.

A full-length codon optimized *BcAA9C* gene was ordered from GenScript Co., and the sequence is submitted in NCBI under GenBank ID-732129. The full-length sequence was used as a template to amplify the catalytic domain (1 to 232 aa), whereas the construct without native linker sequence was designed by either truncating the linker sequence or replacing it with a 21-nucleotide spacer sequence; genes were synthesized from Thermo Fisher Scientific. Specifically, the linker-truncated construct has catalytic (1 to 232 aa) and carbohydrate-binding domains (288 to 321 aa), and a spacer sequence to allow free movement of structured domains. This sequence is submitted in NCBI under GenBank ID-732130. All gene fragments were amplified and digested with EcoRI/NotI restriction enzymes and cloned separately in EcoRI/NotI digested pPICZ(alpha)A vector. The cloning in EcoRI and NotI site in pPICZalpha vector inserts the gene in frame with an N-terminal alpha secretion signal and a C-terminal polyhistidine tag. The cloning was performed in DH5-Alpha and correct insertion of the gene was confirmed via colony PCR using standard AOX (alcohol oxidase) primers provided in the EasySelect Pichia Expression Kit, followed by Sanger sequencing. The positive clones were then linearized using a blunt-end restriction enzyme, SacI, transformed in a chemical-competent P. pastoris KM71H strain, and plated onto a yeast extract peptone dextrose selection plate (10 g/L yeast extract, 20 g/liter Soya Peptone, 20 g/L dextrose,1 M sorbitol, and 20 g/L agar) containing 100 µg/mL Zeocin. Integration in yeast was confirmed by gene-specific PCR, and positive transformants were selected for protein purification.

Protein expression was induced using methanol, with addition of 1% methanol every 24 h, and culture supernatant was harvested after 72 h for protein purification. Briefly, protein secreted in the culture supernatant was precipitated using 60% ammonium sulfate (A4915, Sigma). Following this, precipitate was dialyzed in 20 mM sodium phosphate buffer and 150 mM sodium chloride (pH 8.0), then subjected to Ni-NTA chromatography. The purified enzymes are listed as follows: *fl*^LPMO^ (full-length protein), *cd*^LPMO^ (catalytic domain), and *trunc1*^LPMO^, *trunc2*^LPMO^, and *trunc3*^LPMO^ (linker-truncated chimeric protein) were copper saturated by incubating the enzyme with a 10-fold excess concentration of copper sulfate. The copper was allowed to bind at 25°C for 1 h at 200 rpm. Post-binding, the enzyme was dialyzed against a 20 mM sodium phosphate buffer (pH 6.0) to remove unbound copper.

### *In silico* analysis of stability and structure of CD-CBM chimeric construct.

The physical and chemical properties of linker-truncated fusion proteins (*trunc1*^LPMO^, *trunc2*^LPMO^, *trunc3*^LPMO^) were compared with those of full-length protein (*fl*^LPMO^) using Expasy’s ProtParam server (52). Various parameters analyzed include instability index, aliphatic index, grand average hydropathicity, and half-life of protein. The aliphatic index increased along with the number of aliphatic residues, indirectly indicating an increase in the thermostability of the protein; the instability index measures protein stability, and an index of less than 40 indicates that protein is stable.

The secondary structures of proteins were analyzed using the GOR secondary structure prediction method (53) and the phyre2 online server (54), whereas the 3D structures were modeled using the I-TASSER server (55). The FASTA sequences were submitted and threading was performed using I-TASSER, which generated five models; the one with highest C-score was selected for further analysis. C-score values are typically in a range of −5 to 2 where a higher score reflects a model of better quality (56). Tertiary structures of proteins were verified using the Procheck server (57).

After generating the model, energy minimization was determined by analyzing the 3D structural stability of the chimeric protein using Swiss-PdbViewer (58) and the GROMACS 2020 package with the GROMOS 96, 54A7 force field. The steepest descent method was employed for energy minimization with Particle Mesh Ewald (PME) long-range electrostatics for 50,000 steps (59, 60).

### Enzyme Assays.

An LPMO assay was performed using the protocol described by Ogunmolu et al. (61), with minor modifications. Briefly, the reaction mixture was comprised of 30 µM Ascorbate (Sigma), 50 µM Amplex Red (Sigma), and 7.14 units/mL HRP in a citrate phosphate buffer (pH 6.0). The enzyme assay was performed at 40°C, and 20 µL enzyme was added to 180 µL reaction mix to initiate the reaction. The kinetics was performed for 10 min with 535 nm and 580 nm excitation and emission wavelengths, respectively. The enzyme was excluded in the control reaction. The concentration of H_2_O_2_ released in the LPMO reaction was measured using H_2_O_2_ standard curve (1 to 10 µM). The activity of LPMO was expressed as µmol of H_2_O_2_ released per unit of time.

The cellulose oxidation activities of LPMO enzymes were performed by incubating 10 µM copper-saturated *fl*^LPMO^, *cd*^LPMO^, *trunc1*^LPMO^, *trunc2*^LPMO^, and *trunc3*^LPMO^ with 8 g/L PASC (phosphoric acid swollen cellulose) and 2 mM ascorbate at 45°C for 48 h. The control reaction lacked the enzyme component. Oxidized oligosaccharides were subjected to further hydrolysis by 5 units of beta-glucosidase at 40°C for 24 h. The supernatant was harvested and protein was removed using 20 mM H_2_SO_4_. The release of gluconic acid was estimated using an Agilent 1260 Infinity II HPLC equipped with an Agilent Hi-Plex-H column. The conditions used are as follows: 0.5 mL/min flow rate, column compartment temperature = 30°C, and absorbance 210 nm (62). The concentration of gluconic acid was calculated using a gluconic acid standard curve prepared under similar conditions. The experiment was performed in three biological replicates.

To estimate synergistic depolymerization of complex polysaccharide using LPMO variants and cellulases: we treated 8 g/L PASC with 0.5 FPU commercial enzyme cocktail (C2730; Sigma) and supplemented it separately with 10 µM LPMO variants; for the negative control, LPMO enzymes were excluded. The reaction mix was incubated at 45°C for 72 h at 200 rpm to ensure proper agitation and mixing of reaction content. The supernatant was harvested and analyzed for increases in the concentration of glucose released from biomass treatment, using an Agilent 1260 Infinity II HPLC equipped with an Agilent Hi-Plex Ca column using the following conditions: column temperature = 80°C, refractive index detector temperature = 30°C. Milli-Q water was used as the mobile phase at a flow rate of 0.6 mL/min. The concentration of glucose was calculated against the standard curve prepared by running glucose standard (1 to 10 g/L).

### Binding assays.

The binding of *fl*^LPMO^, *cd*^LPMO^, *trunc1*^LPMO^, *trunc2*^LPMO^, and *trunc3*^LPMO^ enzymes to cellulosic substrates (pretreated biomass and Avicel) was tested by mixing various concentrations of protein (1 to 150 μM) with a fixed substrate concentration. Briefly, 40 g/L water-saturated PTB (80% hydration) was incubated with different concentrations of enzymes in the presence of 20 mM sodium phosphate buffer (pH 6.0) in a total volume of 250 μL. The reaction mixture was incubated in a heat block at 4°C for 12 h at 200 rpm. Protein-bound substrate pellet and unbound proteins in the supernatant were recovered by high-speed centrifugation. The protein in the unbound fraction (supernatant) was estimated using a BCA kit (Pierce, Thermo Fisher Scientific), which was then used to calculate the protein in the bound fraction using the following formula: bound protein = total protein − unbound protein, where total protein indicates the amount of initial protein loaded for the binding experiment. GraphPad Prism 8.0 was used to compute dissociation constant (*K_d_*) and the amount of protein bound at saturation (B_max_). Langmuir binding isotherms were carried out for each protein and bovine serum albumin (BSA) was used as a negative control for the assay. All binding experiments were performed in duplicate.

For SDS-PAGE, 120 μM copper-saturated *fl*^LPMO^, *cd*^LPMO^, *trunc1*^LPMO^, *trunc2*^LPMO^, and *trunc3*^LPMO^ were mixed with 40 g/L (80% water-saturated PTB) and incubated for 12 h at 4°C. Samples were centrifuged and supernatant (unbound), pellet (bound), and total protein fraction were mixed with 6× gel loading dye and run on SDS-PAGE gel. The gel was stained with Coomassie brilliant blue for visualization.

### Thermostability assay.

To determine the thermostability of *fl*^LPMO^, *cd*^LPMO^, *trunc1*^LPMO^, *trunc2*^LPMO^, and *trunc3*^LPMO^, 150 µM copper-saturated enzyme was preincubated at a range of temperatures (35 to 60°C) and the sample was analyzed every 5 min for the presence of residual oxidation activity. Briefly, 50 µL enzyme was incubated for 5 min at gradient temperatures 35, 40, 45, 50, 55, and 60°C in a Bio-Rad thermocycler; postincubation, appropriately diluted LPMO enzymes were analyzed for residual activity by performing an Amplex Red assay. Similarly, five other experimental setups for different time points, i.e., 10, 15, 20, 25, and 30 min, indicated a change in thermostability over time. The release of H_2_O_2_ per unit of time was measured and compared with that of the control (no preincubation). All experiments were done in duplicate.

### Scanning electron microscopy.

The topological changes in plant biomass upon exposure to hydrolytic enzymes was analyzed using scanning electron microscopy (SEM). For this, sodium hydroxide-treated biomass was treated with 10 µM copper-saturated *fl*^LPMO^, *cd*^LPMO^, *trunc1*^LPMO^, *trunc2*^LPMO^, and *trunc3*^LPMO^ with 2 µM ascorbate in 20 µM sodium phosphate buffer (pH 6.0) for 48 h at 45°C. Treatment with commercial enzyme cocktail (SAE0020; Sigma) was used as a positive control, while incubation with water was utilized as a negative control. This differentially treated wheat straw was dried on a coverslip under vacuum in a desiccator. The dried biomass was then carbon-coated and viewed via a scanning electron microscope (EVO 40, Carl Zeiss, Germany). The topology of biomass samples post-treatment was observed by the action of an electron beam at 2 KV, and all samples were analyzed at 20 KX magnification.

### Confocal assay.

Confocal microscopy was used to monitor binding of proteins to PTB. For sample processing, as shown in the binding experiment, 40 g/L (80% water saturated) pretreated biomass was allowed to bind 120 μM *fl*^LPMO^, *cd*^LPMO^, *trunc1*^LPMO^, *trunc2*^LPMO^, and *trunc3*^LPMO^ for 12 h at 4°C under a constant agitation of 200 rpm. For the control, pretreated biomass was incubated with BSA (ab217817, abcam) instead of monooxygenase enzyme. After 12 h, the protein-bound PTB was recovered by high-speed centrifugation. The biomass was washed four times with phosphate-buffered saline (PBS) to remove loosely bound protein. The PTB with bound enzymes (his-tagged) were then incubated with 1:5,000 dilution monoclonal anti-polyhistidine antibody (H1029, Sigma) for 1.5 h at room temperature and washed five times with PBS to remove non-specifically bound antibody. Following this, primary antibody-bound biomass was incubated with 1:15,000 anti-mouse IgG-FITC (fluorescein isothiocyanate) antibody (F0257, Sigma) for 1.5 h in darkness (34). The samples were then extensively washed to remove non-specifically bound antibody. The biomass samples were mounted on the slide with the help of glycerol, and observed on an Olympus BH-2 microscope equipped with epifluorescence irradiation. Confocal microscopy was performed at 492 nm excitation and 520 nm emission maxima, using a Laser Scanning Confocal Microscope (LSM 880, Carl Zeiss). Channel mode visualization was done using 63× Plan-Apochromat Oil immersion DIC (NA 1.40). All experiments were done in triplicate and multiple images were captured for quantitative estimation of binding. The fluorescence was analyzed using open source ImageJ software.
