# Supplementary material for: The Linker Region Promotes Activity and Binding Efficiency of Modular LPMO towards Polymeric Substrate
Source: Microbiol Spectr. 2022 Jan 26;10(1):e02697-21. doi: 10.1128/spectrum.02697-21 (PMC8791183; doi:10.1128/spectrum.02697-21)
Supplement: SUPPLEMENTAL FILE 1 — Supplemental material. Download SPECTRUM02697-21_Supp_1_seq8.pdf, PDF file, 6.4 MB [file spectrum02697-21_supp_1_seq8.pdf]

Supplementary Information

The linker region promotes activity and binding efficiency of modular LPMO towards polymeric substrate

Microbiology Spectrum

Aishwarya Srivastava[1], Pragya Nagar[1], Sumit Rathore[2] and Nidhi Adlakha[1*]

Affiliation Address:

[1]Synthetic Biology and Bioprocessing Group, Regional Centre for Biotechnology, NCR-Biotech Cluster, Faridabad, Haryana, India

[2]Department of Biotechnology, All India Institute of Medical Sciences, New Delhi, India

[*]**Any correspondence should be made to nidhi.adlakha@rcb.res.in**

Table S1 Composition analysis of pretreated biomass

S.No.	Constituent	Composition (%)
**1**	Glucose	68%
**2**	Xylose	11%
**3**	Arabinose	2%

Table S2 Gene Accession ID of *Botrytis cinerea* LPMO belonging to AA9 family

**S.No.**	**Gene**	**NCBI ID**
**1.**	*BcAA9A*	ATZ57781.1
**2.**	*BcAA9B*	ATZ50474.1
**3.**	*BcAA9C*	ATZ50551.1
**4.**	*BcAA9D*	ATZ51291.1
**5.**	*BcAA9E*	ATZ48364.1
**6.**	*BcAA9F*	ATZ55262.1
**7.**	*BcAA9G*	ATZ53918.1
**8.**	*BcAA9H*	ATZ55836.1
**9.**	*BcAA9I*	ATZ46857.1

1. fl^{LPMO}

MKLQLIIPFSFLISYVSAHTIFMKLQSGGTLYNTSYAIRTPTYDGPINDVTTEYVACNGGPNPT
TPSSNIINVVAGSTVNAIWRHTLDSTPANDATYVLDPSHLGPVMAYMKKVTDATTDVGYGPGWF
KISEQGLNVATQGWATTDLINNAGVQSITIPSCIANGQYLLRAELIALHSAGGSQGAQLYMECA
QINVSGGTGTSTPSTVSFPGAYGQSNPGILINIYQTLTTYTIPGPTPFVCGAAQSTAKSSSTST
AKPTSTSTLSTSTVTKTSSSAVASGTGTAAIYAQCGGQGWTGATVCASGSKCVVSSAFYSQCLP
S

2. trunc1^{LPMO}

MKLQLIIPFSFLISYVSAHTIFMKLQSGGTLYNTSYAIRTPTYDGPINDVTTEYVACNGGPNPT
TPSSNIINVVAGSTVNAIWRHTLDSTPANDATYVLDPSHLGPVMAYMKKVTDATTDVGYGPGWF
KISEQGLNVATQGWATTDLINNAGVQSITIPSCIANGQYLLRAELIALHSAGGSQGAQLYMECA
QINVSGGTGTSTPSTVSFPGAYGQSNPGILINIYQTLTTYTIPGPTPFVCGAAQSTAKYAQCGG
QGWTGATVCASGSKCVVSSAFYSQCLPS

3. trunc2^{LPMO}

MKLQLIIPFSFLISYVSAHTIFMKLQSGGTLYNTSYAIRTPTYDGPINDVTTEYVACNGGPNPT
TPSSNIINVVAGSTVNAIWRHTLDSTPANDATYVLDPSHLGPVMAYMKKVTDATTDVGYGPGWF
KISEQGLNVATQGWATTDLINNAGVQSITIPSCIANGQYLLRAELIALHSAGGSQGAQLYMECA
QINVSGGTGTSTPSTVSFPGAYGQSNPGILINIYQTLTTYTIPGPTPFVCGAAQSTAKSSSTST
AKPTSTSTLSTSTVTKTSSSYAQCGGQGWTGATVCASGSKCVVSSAFYSQCLPS

4. trunc3^{LPMO}

MKLQLIIPFSFLISYVSAHTIFMKLQSGGTLYNTSYAIRTPTYDGPINDVTTEYVACNGGPNPT
TPSSNIINVVAGSTVNAIWRHTLDSTPANDATYVLDPSHLGPVMAYMKKVTDATTDVGYGPGWF
KISEQGLNVATQGWATTDLINNAGVQSITIPSCIANGQYLLRAELIALHSAGGSQGAQLYMECA
QINVSGGTGTSTPSTVSFPGAYGQSNPGILINIYQTLTTYELNYFQGYAQCGGQGWTGATVCAS
GSKCVVSSAFYSQCLPS

Fig. S1 Sequence of *BcAA9C* gene, where catalytic domain, linker sequence and carbohydrate binding module are highlighted green, yellow and blue respectively

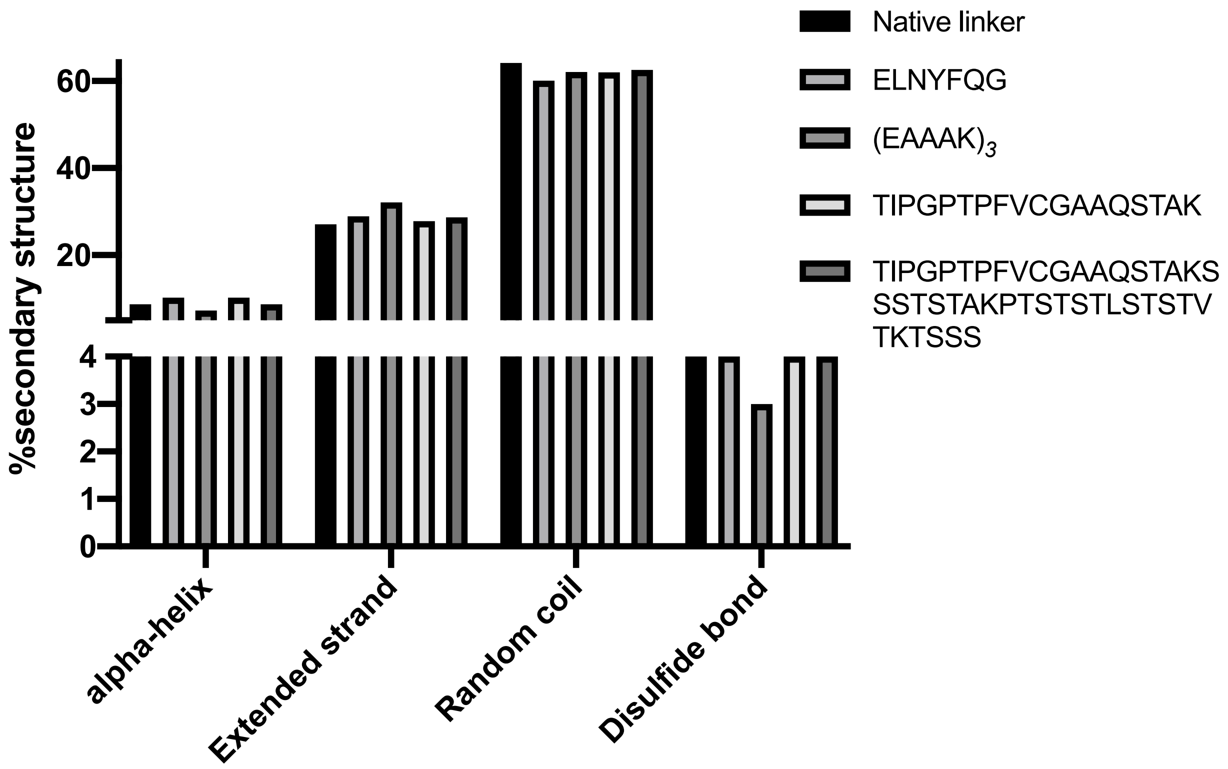

Fig. S2 Secondary structure of *fl*^LPMO and CD-CBM chimeric variants. GOR server uses primary amino acid sequence as input whereas pdbsum takes modelled pdb structure as input to predict the secondary structure. The graph indicated the truncated constructs demonstrated disulfide bond pattern, alpha-helix and beta-strand secondary structure similar to native linker.

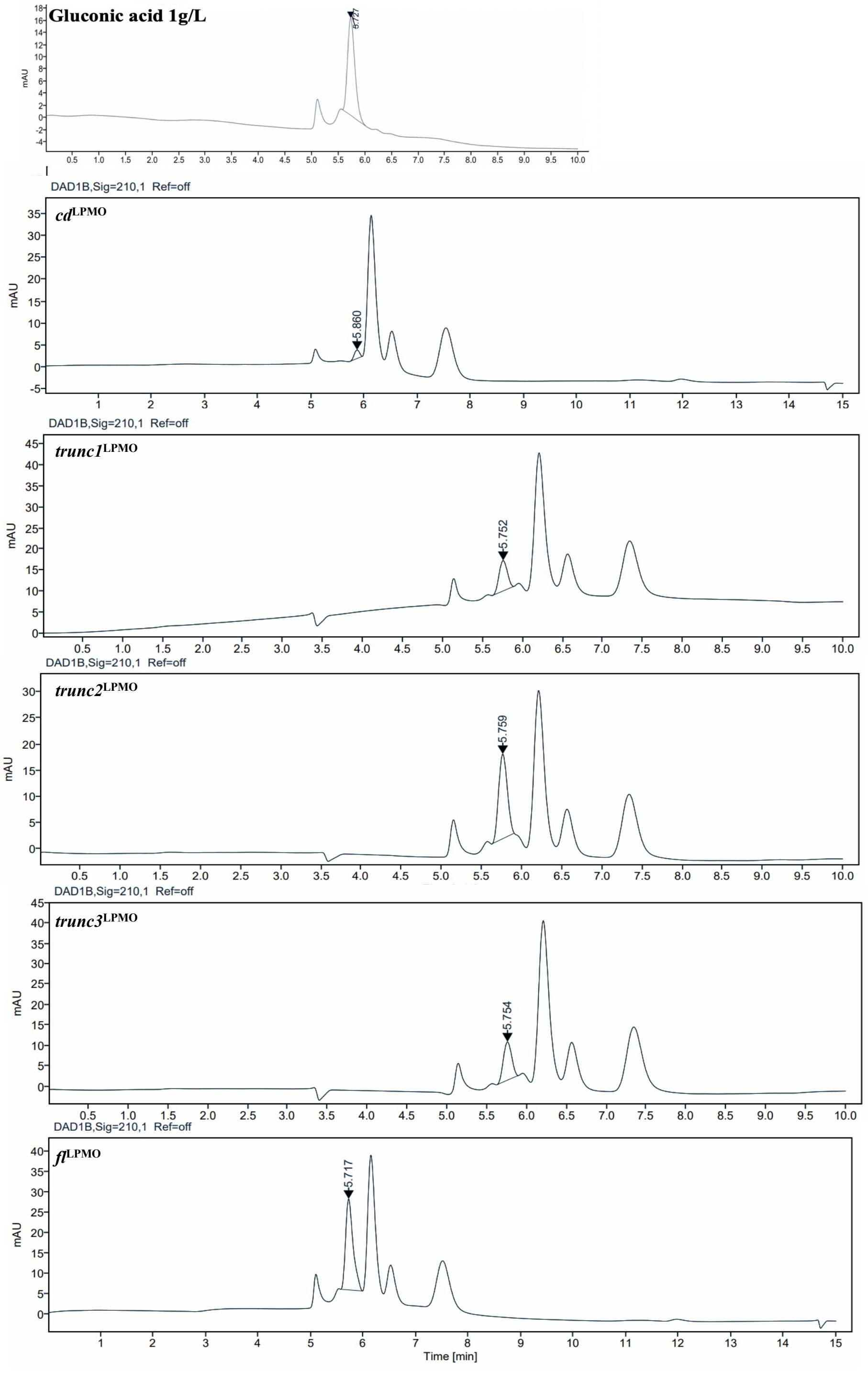

Fig. S3 HPLC profile of PASC treated with LPMO variants. 8 g/L PASC was treated with 10μM copper saturated LPMO variants. The reaction mix was treated with glucosidase and release of gluconic acid was measured using HPLC equipped with PDA at 210 nm.

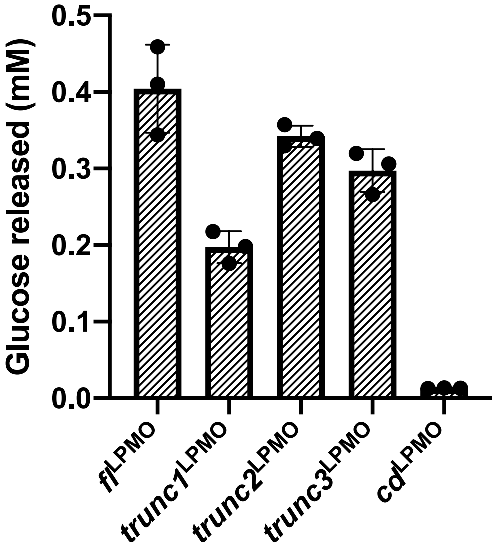

Fig. S4 Effect of LPMO treatment on release from biomass. The pretreated biomass was incubated with 10µM *fl*^{LPMO}, *trunc1*^{LPMO}, *trunc2*^{LPMO}, *trunc3*^{LPMO} and *cd*^{LPMO} separately for 48 h at 45°C. The release of glucose post enzyme treatment was measured using GOD-POD kit (GAGO20, Sigma). Briefly, 200 µl Assay reagent was added to 100 µl clear supernatant. The reaction was incubated at 37°C for 30 min. The reaction was stopped using 200 µl 6 N H_2SO_4 and read at 540 nm. Assay reagent was not added to the control reaction. Error bar represent standard deviation of three independent experiments.

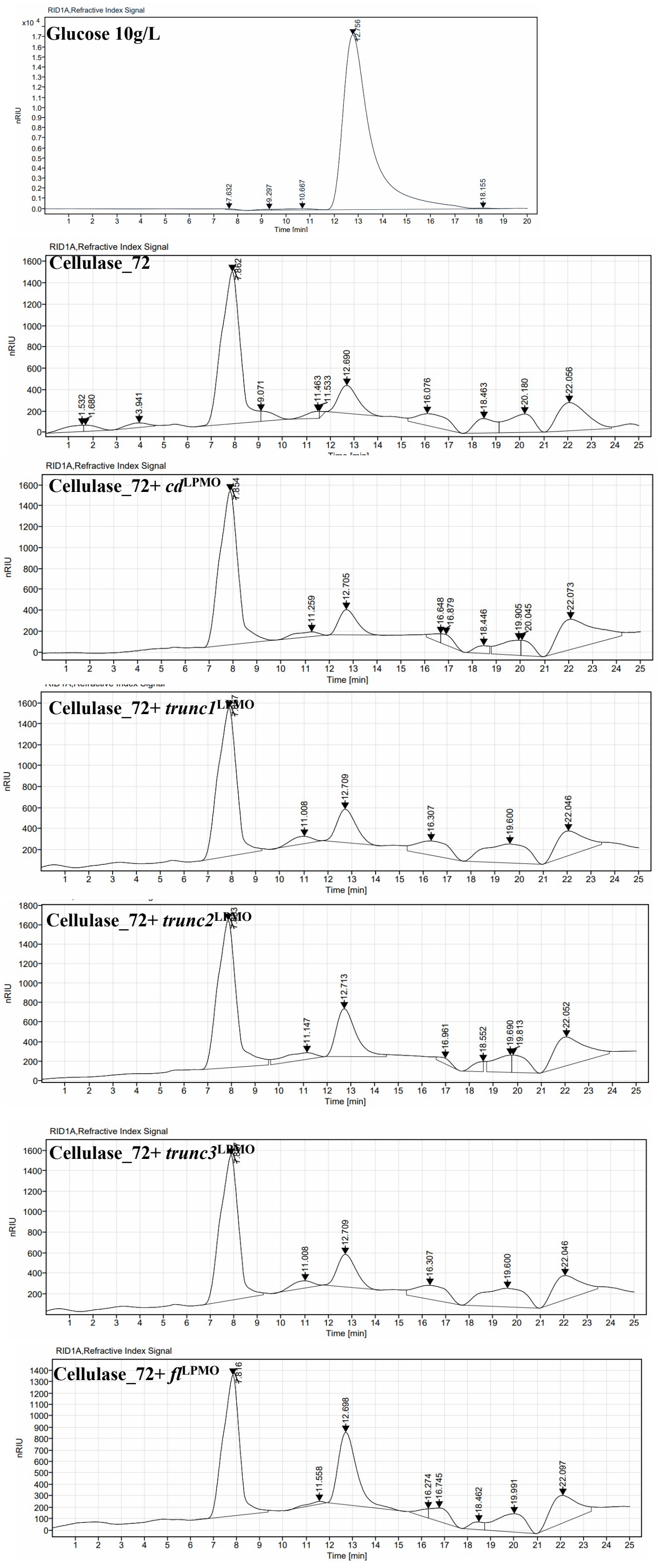

Fig. S5 HPLC profile of PASC treated with LPMO variants. 8 g/L PASC was treated with 0.5 FPU cellulase for 72 h and 10 μM copper saturated LPMO variants are supplemented. The

amount of glucose released was evaluated using HPLC equipped HiPlex calcium and the effect of supplementation was calculated.

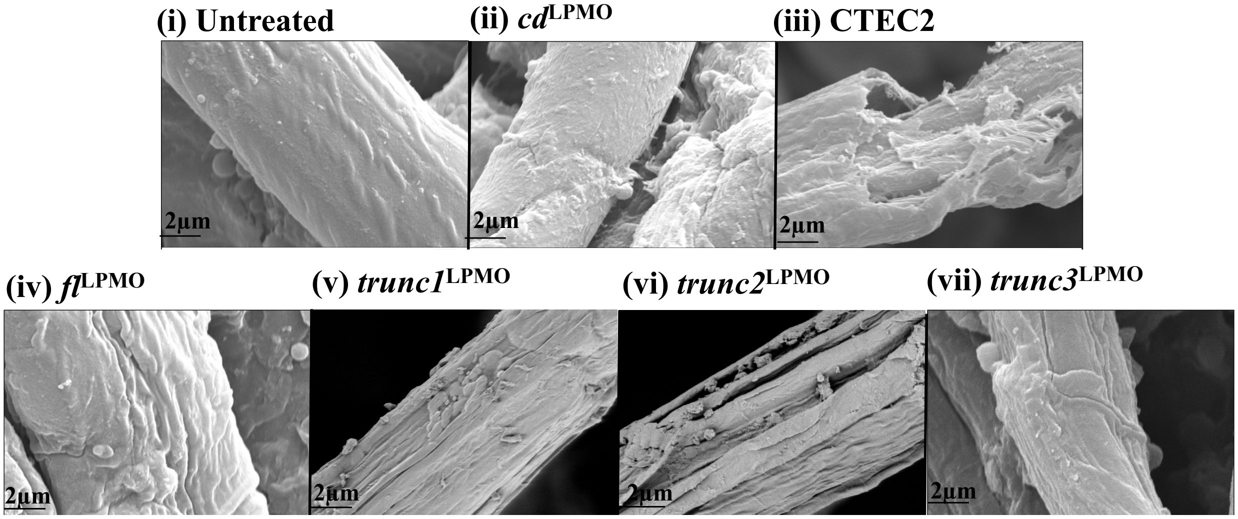

Fig. S6 LPMO-assisted biomass degradation (a) Enzyme mediated change in biomass structure was visualized using Scanning Electron Microscopy (SEM). The pretreated biomass was incubated with 10 µM copper saturated *fl*^{LPMO}, *trunc1*^{LPMO}, *trunc2*^{LPMO}, *trunc3*^{LPMO}and *cd*^{LPMO} separately for 48 h at 45°C.
